# The Obesity Paradox in Lung Cancer: Associations With Body Size Versus Body Shape

**DOI:** 10.3389/fonc.2020.591110

**Published:** 2020-11-10

**Authors:** F. H. Ardesch, R. Ruiter, M. Mulder, L. Lahousse, B. H. C. Stricker, J. C. Kiefte-de Jong

**Affiliations:** ^1^Department of Epidemiology, Erasmus Medical Center, Rotterdam, Netherlands; ^2^Department of Public Health and Primary Care/LUMC Campus The Hague, Leiden University Medical Center, The Hague, Netherlands; ^3^Department of Bioanalysis, Faculty of Pharmaceutical Sciences, Ghent University, Ghent, Belgium

**Keywords:** waist-to-hip ratio (WHR), waist circumference (WC), body mass index (BMI), A Body Shape Index (ABSI), lung cancer risk, obesity

## Abstract

**Background:**

The association between obesity and lung cancer (LC) remains poorly understood. However, other indices of obesity on the basis of body shape instead of body size have not been examined yet. The aim of this study was to evaluate the association between different indices of body size and body shape and the risk of LC. In particular, this study examined the association between A Body Shape Index, a more precise indicator of abdominal fat than traditional anthropometric measures, and the risk of LC.

**Methods:**

In the prospective cohort the Rotterdam Study, we analysed data of 9,689 participants. LC diagnoses were based on medical records and anthropometric measurements were assessed at baseline. Cox-regression analyses with corresponding Hazard Ratios were used to examine the association between the anthropometric measurements and the risk of LC with adjustment for potential confounders. Potential non-linear associations were explored with cubic splines using the Likelihood ratio (LR) test.

**Results:**

During follow-up, 319 participants developed LC. Body mass Index (BMI) was inversely associated with the risk of lung cancer (HR 0.94, 95% CI: 0.91–0.97) and persisted after excluding lung cancer cases during the first 10 years of follow-up. There was evidence for a non-linear association between BMI and the risk of lung cancer (0,04, df = 1), which indicated that the inverse association between BMI and lung cancer was mainly present in non-obese participants. Waist circumference (WC) (HR 1.03 95% CI: 1.01–1.05), Waist-to-Hip Ratio (WHR) (HR 1.23 95% CI: 1.09–1.38) and ABSI (A Body Shape Index) (HR 1.17 95% CI: 1.05–1.30) were positively and linearly associated with the risk of lung cancer.

**Conclusions:**

Body shape rather than body size may be an important risk indicator of LC. Future research should focus on the role of visceral fat and the risk of LC as well as the underlying mechanisms.

## Introduction

Lung cancer is currently the main cause of cancer mortality worldwide (1). In 2018, the number of incident lung cancer cases was approximately 2.9 million worldwide, accompanied by 1.75 million cases of death by lung cancer (2, 3). Apart from smoking, emerging evidence shows that obesity may play a role in the development of cancer. Although obesity is associated with 13 types of cancer (4), the association between obesity and lung cancer remains controversial and the underlying mechanisms are poorly understood.

Several indices of nutritional status exist. The most commonly used method is Body Mass Index (BMI) due to its simplicity of measuring and low cost (5). Several meta-analyses have shown an inverse association between BMI and the risk of lung cancer (6–9). A first explanation may come from residual confounding by smoking. Multiple studies that assessed the role of residual confounding by restricting the association between BMI and the risk of lung cancer to never smokers were not able to fully explain the inverse association between BMI and lung cancer (9–13). A second explanation for this inverse association may be due to weight loss as a result of preclinical lung cancer or an impaired lung function e.g. by chronic obstructive lung disease (14). It has been suggested that the association of BMI and lung cancer should increase to a more positive association after excluding lung cancer cases during the first years of the follow-up which may include imminent cases of lung cancer. However, several studies that excluded lung cancer cases in the first years of follow-up have found inconsistent results (9, 15–20).

In contrast to overall obesity and body size, as measured by BMI, specifically abdominal obesity may have a more important role in lung cancer development (9, 21–23). Abdominal obesity as a measure of body shape can be reflected by an increased Waist-to-Hip Ratio (WHR) and Waist circumference (WC), independent of BMI (24, 25). A meta-analysis, including seven prospective cohorts, has showed that WC and WHR were positively associated with the risk of lung cancer (21). However, this meta-analysis consisted of mainly women and measurements were mostly self-reported and/or self-measured. Meanwhile, more recent studies with larger sample sizes have shown more controversial results; a pooled analysis of 12 cohorts has concluded that both WC and WHR were positively associated with the risk of lung cancer (9), whereas another prospective cohort study has found an inverse association for WC and the risk of lung cancer in Chinese participants (23), and a study among 162.679 American adults has found no association between BMI and WC with the risk of lung cancer (22).

A newly developed anthropometric measurement is A Body Shape Index (ABSI), combining WC to BMI and weight into one formula (26). Studies have shown that ABSI is an independent predictor of the fat and fat free mass ratio (27), indicating that ABSI may be a more precise indicator of abdominal fat. Moreover, a meta-analysis has shown that ABSI outperformed BMI and WC in predicting all-cause mortality including cancer (28). However, to the best of our knowledge, the association between ABSI and the risk of lung cancer has not been examined yet.

The objective of this study was to evaluate the different indices of body size (i.e. BMI) and body shape (i.e. WC, WHR, and ABSI) and the risk of lung cancer in a population-based prospective cohort study.

## Methods

### Study Design

Data for this prospective cohort study was derived from three sub cohorts from the ongoing prospective Rotterdam Study (29). The Rotterdam study started its first cohort in 1990 (RS-I) and has expanded with two additional cohorts in 2000 (RS-II) and 2006 (RS-III), consisting of participants living in the district of Ommoord in the city of Rotterdam. At the start of each cohort and subsequently every 3 to 4 year, all participants were examined in detail. Participants were interviewed at their home (2 h) and afterwards, trained staff exposed these participants to multiple measurements in a special research facility centre (5 h). An overview of the various cohorts with the various follow-up measurements and starting dates were described elsewhere (29). A written Informed consent was signed by all participants. The institutional review board of the Erasmus MC as well as the review board of The Netherlands Ministry of Health Welfare and Sports approved the Rotterdam Study (29).

### Study Population and Exclusion Criteria

The population in this study consisted of participants from the first three cohorts that started in 1990 (RS-I), 2000 (RS-II), and in 2006 (RS-III). By 2008, the total number of participants in the Rotterdam Study at baseline consisted of 14,926 participants aged 45 or over. In this study we excluded participants without all investigated anthropometric or lifestyle measurements at baseline from the initial cohort (N= 5,237). Therefore, the number of participants at baseline was equal to 9,689. All participating women were postmenopausal.

### Assessment of Lung Cancer

The primary outcome of interest was incident lung cancer. Two research physicians independently assessed the diagnosis of cancer based on medical records obtained through general practitioners and hospital discharge letters. Additional information was collected through linkage with the Dutch Hospital Data, National Cancer Registry, and Dutch pathology database (PALGA). Only cases confirmed by pathology were used. Cases of mesothelioma were not included in the definition of cancer for these analyses. Lung cancer was classified according to the International Classification of Diseases tenth edition (C34.2, C34.3). In case of discrepancy, consensus was obtained through consultation with a cancer epidemiologist. Follow-up of cancer registration was completed up to January 1, 2014.

### Anthropometric Measurements

The anthropometric measurements Body Mass Index (BMI), Waist circumference (WC), Waist-to-Hip Ratio (WHR), and A Body Shape Index (ABSI) were measured in the research centre by trained staff during regular on site visits every 3–4 years.

BMI (kg/m^2^) was calculated by dividing the weight (kg) by the squared height (m^2^). Height and weight were measured without shoes and heavy outerwear, using calibrated SECA-measurement scales. BMI was categorised as normal, overweight, and obese with the corresponding cut offs <25, 25–30, and >30 kg/m², respectively (24). Since the underweight class (<18.5 kg/m²) contained only five lung cancer cases, we merged this class with the normal weight class to warrant the sample size.

WC (cm) was measured with a flexible measuring tape at the level halfway between the lower rib margin and the iliac crest in standing position, without heavy outerwear, empty pockets, and calm exhaling. According to recommendations of the WHO (World Health Organization), WC was categorised as normal, increased, and substantially increased with the corresponding cut offs <80, 80–88, and >88 cm, respectively, in females and <94, 94–102, and >102 cm, respectively, in males (24). **

WHR was defined as the WC (in cm) divided by the Hip Circumference (HC). HC (in cm) was measured as the maximum circumference over the nates. According to recommendations of the WHO, WHR was categorised as normal, increased, and substantially increased with the corresponding cut offs <0.80, 0.80–0.85, and >0.85 respectively, in females and <0.95, 0.95–1.00 and >1.00, respectively, in males (24). We standardised the continuous WHR values into age (continuous) and sex adjusted z-scores, specific to the sample of analysis.

ABSI (A Body Shape Index) was calculated as WC/(BMI^2/3^×height^1/2^) (26). To compare low ABSI-values with higher ABSI-values, we divided ABSI into tertiles. As with WHR, we standardised the continuous ABSI values into age (continuous) and sex adjusted z-scores, specific to the sample of analysis.

### Covariate Assessment

Covariates of interest were derived from the existing literature and measured at baseline. We considered the following covariates as important due to their relation with the anthropometric measurements and the risk of lung cancer: age; employment; highest educational level; marital status; sex; ever diagnosed for cancer; smoking status; pack years; physical activity; WCRF/AICR (World Cancer research Fund/American Institute for Cancer Research) adherence score; alcohol consumption.

Paid employment (yes/no), educational level (high: higher general education or university/low: primary education or intermediate vocational), marital status, (partner/no partner) and sex (men/women) ever diagnosed for cancer (yes/no) and smoking status (never, ever, current), together with the corresponding cigarettes in pack years (continuously), were self-reported during the home interviews. Physical activity (30, 31) was measured in MET-hours and assessed with an adapted version of the Zutphen Physical Activity questionnaire (32). For the current analyses, we standardised the continuous MET hours per week values into z-scores, specific to the sample of analysis. The WCRF/AICR adherence score contained five recommendations for cancer prevention including: avoiding energy-dense foods, sugar-containing drinks, red and processed meat, and stimulating fruit and vegetables, dietary fiber, and vitamin supplementation. A minimum sum score of 0 points indicated that none of the recommendations was met and a maximum sum score of 5 indicated that all recommendations were fully met. Dietary intake items as well as alcohol consumption (grams of ethanol per day) were measured by using the validated food frequency questionnaire (FFQ) (33).

### Statistical Analysis

We used Cox proportional hazards regression to analyse the association between BMI, WC, WHR, and ABSI values with the risk of lung cancer. Both continuous and categorical analyses were performed. For all categorical variables, we used the lowest category as reference category. Results of the Cox-regression analyses were presented as hazard ratios (HRs) with the corresponding 95% confidence intervals (CI). The underlying time axis of the Cox-regression model was the follow-up time, starting at baseline until the first diagnosis of lung cancer, death, or end of the study period, whichever came first. Results of the Cox-regression analysis were presented in a crude model (model 1) which included the association between the main determinant and the outcome variable, adjusted for sex and age, as well as a multivariable model, adjusting the association for all (potential) confounders at baseline (model 2). Furthermore, we used the Wald-test to calculate the overall p-for trend for the categorical variables. We added an interaction term between the covariates and the underlying follow-up time to test for the proportional hazard assumption. In addition to the baseline measurements, we analysed the mean differences between baseline and follow-up of BMI, WC, WHR, and ABSI in relation to the risk of lung cancer to examine whether the association might change over time. Within these associations, the baseline measurement of each anthropometric measurement was added to account for potential regression to the mean (34). These mean differences were calculated by using a paired sample t-test.

We tested for effect modification by sex (men/women), smoking status (never, ever current), and pack years (continuous). We added an interaction term between the main determinant(s) and sex (determinant*sex) in an unadjusted Cox-regression model. Whenever the interaction term was significant, the results were presented categorized by sex, smoking status or stratified by the median of pack years.

In this study, we performed two sensitivity analyses. First, we systemically excluded lung cancer cases within the first 5, 7.5, and 10 years of follow-up to examine whether reverse causation could influence the association between BMI at baseline and the risk of lung cancer. Second, to assess the role of residual confounding by smoking, the association between BMI and the risk of lung cancer was stratified for never, ever and, current smokers.

The possibility of non-linear associations between the anthropometric measurements BMI, WC, WHR and, ABSI with the risk of lung cancer were assessed by using data driven natural cubic splines as suggested by Durrleman et al. (34). Degrees of freedom were determined on the basis of the lowest Akaike’s information criterion (AIC) value. Likelihood ratio tests (LRTs) were performed to ascertain whether a non-linear model suited the data better than a linear model. Whenever the LRTs showed a significant result, the effect estimates were separated for the different intervals. The cut-offs of the interval were then based on the knots in the cubic spline.

All missing values, except for the outcome variable, were imputed by using the Markov chain Monte Carlo (MCMC) method. After the imputation procedure, the resulting ten datasets were pooled and an average of the imputed data was calculated. The pooled data was used to conduct all analyses in this study. In this study, p-values less than 0.05 (two-sided) were considered as statistically significant. For the non-linear Likelihood-ratio (LR) test we used R-statistics version 4.4.3. In all further statistical analyses we used Statistical Product and Service Solutions (SPSS) version 24.0.

## Results

### Study Population Characteristics

**Table 1** shows the main baseline characteristics of the study population (n= 9,689). Within a mean follow-up of 13.2 years, 319 participants developed lung cancer. Most participants developed lung cancer in cohort 1 (88.7%) within a mean follow-up of 16.0 years. According to the guidelines of the WHO, most of the participants showed a substantially increased WC in all cohorts. Similarly, this trend was also observed for WHR, except for RS-III. Participants in RS-III showed the lowest age, WHR, ABSI, but the highest BMI on average.

**Table 1:** Baseline characteristics by cohort in the Rotterdam Study.

Characteristic	Total baseline, n = 9689	RS-I, n = 5397	RS-II, n =1616	RS-III, n = 2676	P-for trend
**Follow-up (years) mean ± SD**	13.2 (6.8)	16.0 (7.0)	13.5 (3.4)	7.3 (1.4)	<0.001
**Sex, *n* (%)**					
Men	4082 (42.1)	2217 (41.0)	743 (46.0)	1122 (41.9)	0.002
Women	5607 (57.9)	3180 (59.0)	873 (54.0)	1554 (58.1)	
**Age (years), mean ± SD**	64.1 ± 8.7	67.7 ± 7.8	63.8 ± 7.3	57.1 ± 6.6	<0.001
**Body Mass Index (kg/m^2^), mean ± SD**	26.8 ± 4.0	26.3 ± 3.6	27.3 ± 4.1	27.5 ± 4.5	<0.001
**Body Mass Index^a^, *n* (%)**					
Normal weight	3374 (34.9)	2068 (38.3)	478 (29.6)	828 (30.9)	<0.001
Overweight	4594 (47.4)	2560 (47.4)	795 (49.2)	1239 (46.3)	
Obese	1721 (17.7)	769 (14.3)	343 (21.2)	609 (22.8)	
**Waist circumference (cm), mean ± SD**	91.6 ± 11.8	90.2 ± 11.0	94.0 ± 11.9	93.1 ± 11.8	<0.001
**Waist circumference^b^, *n* (%)**					
Normal risk	2993 (30.9)	1856 (34.4)	400 (24.8)	737 (27.5)	<0.001
Increased risk	2920 (30.1)	1617 (30.0)	513 (31.7)	790 (29.5)	
Substantially increased risk	3776 (39.0)	1924 (35.6)	703 (43.5)	1149 (43.0)	
**Waist-to-Hip Ratio, mean ± SD**	0.895 (0.092)	0.903 (0.092)	0.911 (0.091)	0.868 (0.086)	<0.001
**Waist-to-Hip Ratio^c^, *n* (%)**					
Normal risk	3468 (35.8)	1704 (31.6)	466 (28.8)	1298 (48.5)	<0.001
Increased risk	2466 (25.5)	1344 (24.9)	442 (27.4)	680 (25.4)	
Substantially increased risk	3755 (38.7)	2349 (43.5)	708 (43.8)	698 (26.1)	
**ABSI, mean ± SD**	0.079 (0.0058)	0.080 (0.0063)	0.080 (0.0054)	0.0785(0.0060)	<0.001
**ABSI, *n* (%)**					
Tertile 1	3229 (33.3)	1896 (35.1)	412 (25.5)	921 (34.4)	<0.001
Tertile 2	3230 (33.3)	1631 (30.2)	552 (34.2)	1047 (39.1)	
Tertile 3	3230 (33.3)	1870 (34.7)	652 (40.3)	708 (26.5)	
**Physical activity (MET hours), median (IQR)**	-0.170 (1.22)	-0.150 (1.3)	-0.106 (1.2)	-0.269 (1.1)	0.135
**Alcohol intake (g/day), median (IQR)**	5.29 (17.3)	3.45 (14.7)	8.39 (21.1)	7.95 (18.3)	<0.001
**Smoking status, *n* (%)**					
Never smoker	3117 (32.2)	1814 (33.6)	470 (29.0)	833 (31.1)	0.002
Ever smoker	4279 (44.2)	2327 (43.1)	771 (47.7)	1181 (44.1)	
Current smoker	2293 (23.6)	1256 (23.3)	375 (23.3)	662 (24.8)	
**Pack-years, median (IQR)**					
Ever smoker	15.0 (26.6)	18.7 (31.2)	13.3 (26.8)	11.3 (21.1)	<0.001
Current smoker	26.3 (29.4)	30.0 (26.8)	20.7 (37.3)	20.5 (34.8)	<0.001
**Education, *n* (%)**					
High	4207 (43.4)	1981 (36.7)	863 (53.4)	1473 (55.0)	<0.001
Low	5482 (56.6)	3416 (63.3)	753 (46.6)	1203 (45.0)	
**Employed, *n* (%)**					
Yes	2696 (27.8)	666 (12.3)	394 (24.4)	1636 (61.1)	<0.001
No	6993 (72.2)	4731 (87.7)	1222 (75.6)	1040 (38.9)	
**Partner, *n* (%)**					
Yes	7090 (73.2)	3744 (69.4)	1230 (76.1)	2116 (79.1)	<0.001
No	2599 (26.8)	1653 (30.6)	386 (23.9)	560 (20.9)	
**WCRF adherence, mean ± SD** **DM, *n* (%)**	3.54 (0.8)	3.48 (0.8)	3.77 (0.8)	3.51 (0.8)	<0.001
Yes	1226 (12.7)	781 (14.5)	203 (12.6)	242 (9.0)	<0.001
No	8463 (87.3)	4616 (85.5)	1413 (87.4)	2434 (91.0)	
**Ever diagnosed for cancer, *n* (%)**					
Yes	1448 (15.0)	1015 (18.8)	195 (12.0)	278 (10.4)	<0.001
No	8241 (85.0)	4382 (81.2)	1421 (88.0)	2438 (89.6)	

LC, lung cancer; SD, Standard Deviation; IQR, Interquartile Range; BMI, Body Mass Index; WC, Waist circumference; WHR, Waist-to-Hip Ratio; ABSI, A Body Shape Index; MET, Metabolic Equivalents; WCRF, World Cancer Research Fund; DM, Diabetes Mellitus.

a BMI was categorised as normal, over weighted, and obese with the corresponding cut offs <25, 25–30, and >30, respectively.

b Waist circumference was categorised as normal, increased, and substantially increased with the corresponding cut offs <80, 80-88, and >88 cm, respectively, in females and <94, 94-102, and >102 cm, respectively, in males.

c Waist-to-Hip-Ratio was categorised as normal, increased, and substantially increased with the corresponding cut offs <0.80, 0.80-0.85, and >0.85, respectively, in females and <0.95, 0.95-1.00, and >1.00, respectively, in males.

### Anthropometric Measurements at Baseline and the Risk of Lung Cancer

As shown in **Table 2**, an 1 kg/m^2^ increase of BMI was associated with a reduced risk of lung cancer, after adjustment for sex and age (HR 0.95, 95% CI: 0.92–0.98) and after full adjustment for sex, age, cohort, physical activity, alcohol intake, pack-years, education, WCRF adherence score, and diabetes mellitus (HR 0.94, 95% CI: 0.91–0.97). This corresponding inverse trend was also observed for the normal BMI group (reference group, BMI<25) compared with the high and obese group, after adjustment for sex and age (High: HR 0.76 95% CI: 0.60–0.96) (Obese: HR 0.68 95% CI: 0.47–0.99) and in the fully adjusted model (High: HR 0.72 95% CI: 0.56–0.91) (Obese: HR 0.61 95% CI: 0.41–0.89). Furthermore, 1 cm of WC (HR 1.03 95% CI: 1.01–1.06) and one standard deviation increase of WHR (HR 1.23 95% CI: 1.09–1.38) and ABSI (HR 1.17 95% CI: 1.05–1.30) were positively associated with a 3%, 23%, and 17% higher risk of lung cancer respectively, after full adjustment for confounders. Similar results were observed when WHR (HR 1.66 95% CI: 1.21–2.26, P-for trend = 0.06) and ABSI (HR 1.50 95% CI: 1.05–2.12, P-for trend = <0.001) were analyzed categorically.

**Table 2:** Hazard ratio’s (95% CI) for the association between BMI (kg/m^2^), WC (cm), WHR, and ABSI with the risk of lung cancer.

Anthropometric measurement	Number of LC cases	Model 1 HR, 95% CI	Model 2 HR, 95% CI
**Body Mass Index^a^ (kg/m^2^)**			
Normal	138	1.00 Referent	1.00 Referent
High	145	0.76 (0.60–0.96)*	0.72 (0.56–0.91)*
Obese *P-trend*	36	0.68 (0.47–0.99)*0.009	0.61 (0.41–0.89)*0.037
Continuous	319	0.95 (0.92–0.98)*	0.94 (0.91–0.97)*
**Waist circumference^b^ (cm)**			
Normal risk	123	1.00 Referent	1.00 Referent
Increased risk	95	0.89 (0.68–1.18)	0.99 (0.73–1.36)
Substantially increased risk *P-trend*	101	0.94 (0.71–1.24)*0.0676*	1.22 (0.88–1.78)*0.652*
Continuous	319	1.02 (1.00–1.04)*	1.03 (1.01–1.05)*
**Waist-to-Hip Ratio^c^**			
Normal risk	96	1.00 Referent	1.00 Referent
Increased risk	83	1.28 (0.95–1.74)	1.32 (0.97–1.80)
Substantially increased risk *P-trend*	140	1.58 (1.18–2.11)*0.421	1.66 (1.21–2.26)*0.0625
Continuous	319	1.18 (1.07–1.31)*	1.23 (1.09–1.38)*
**ABSI**			
Tertile 1	70	1.00 Referent	1.00 Referent
Tertile 2	106	1.26 (0.92–1.72)	1.38 (0.98–1.95)
Tertile 3 *P-trend*	143	1.56 (1.06–2.01)*0.000	1.50 (1.06–2.12)*0.016
**Continuous**	319	1.19 (1.09–1.31)*	1.17 (1.05–1.30)*

HR, Hazard Ratio; CI, Confidence Interval; LC, lung cancer; ABSI, A Body Shape Index.

Model 1: Adjusted for sex and age.

Model 2: Adjusted for sex, age, cohort (RS-I/RS-II/RS-III), physical activity (continuous), alcohol intake (continuous), pack-years (continuous), education (high/low), WCRF adherence score (continuous), diabetes mellitus (yes/no). WC and WHR were additionally adjusted for BMI.

^a^BMI was categorised as normal, over weighted, and obese with the corresponding cut offs <25, 25–30, and >30, respectively.

^b^Waist circumference was categorised as normal, increased, and substantially increased with the corresponding cut offs <80, 80-88, and >88 cm, respectively, in females and <94, 94–102, and >102 cm, respectively, in males.

^c^Waist-to-Hip-Ratio was categorised as normal, increased, and substantially increased with the corresponding cut offs <0.80, 0.80-0.85, and >0.85, respectively, in females and <0.95, 0.95–1.00, and >1.00, respectively, in males.

*Underlined values indicate a statistically significant association.

### Sensitivity Analyses

**Table 3** shows that the inverse association between BMI and the risk of lung cancer persisted after excluding lung cancer cases in the first 5, 7.5, and 10 years of follow-up, before and after full adjustment for sex, age, cohort, physical activity, alcohol intake, pack-years, education, WCRF adherence score, and diabetes mellitus. Moreover, in both models, the inverse association remained stable as the number of excluded follow-up years increased. **Table 4** shows the associations between BMI and the risk of lung cancer, stratified for never, ever, and current smokers. Before and after full adjustment for confounders, BMI was not associated with the risk of lung cancer in never, ever, and current smokers.

**Table 3:** Hazard ratio’s (95% CI) for BMI (kg/m^2^) with the risk of lung cancer after excluding lung cancer cases during the first 5, 7.5, and 10 years of follow-up after baseline measurement.

	Number of LC cases	Model 1 HR, 95% CI	Model 2 HR, 95% CI
**Excluded follow-up years**			
5	245	0.95 (0.91–0.99)*	0.94 (0.90–0.98)*
7.5	204	0.95 (0.91–0.99)*	0.94 (0.90–0.98)*
10	162	0.95 (0.91–1.00)*	0.95 (0.90–0.99)*

HR, Hazard Ratio; CI, Confidence Interval; LC, Lung Cancer.

Model 1: Adjusted for sex and age.

Model 2: Adjusted for sex, age, cohort (RS-I/RS-II/RS-III), physical activity (continuous), alcohol intake (continuous), pack-years (continuous), education (high/low), WCRF adherence score (continuous), and diabetes mellitus (yes/no).

*Underlined values indicate a statistically significant association.

**Table 4:** Hazard ratio’s (95% CI) for BMI (kg/m^2^) and the risk of lung cancer in never, ever and current smokers.

	Number of LC cases	Model 1 HR, 95% CI	Model 2 HR, 95% CI
Never smokers	33	0.97 (0.90–1.07)	0.97 (0.89–1.07)
Ever smokers	102	0.99 (0.93–1.06)	0.96 (0.89–1.03)
Current smokers	167	0.98 (0.93–1.02)	0.98 (0.94–1.03)

HR, Hazard Ratio; CI, Confidence Interval; LC, lung cancer; ABSI, A Body Shape Index.

Model 1: Adjusted for sex and age.

Model 2: Adjusted for sex, age, cohort (RS-I/RS-II/RS-III), physical activity (continuous), alcohol intake (continuous), pack-years (continuous), education (high/low), WCRF adherence score (continuous), and diabetes mellitus (yes/no).

### Anthropometric Mean Differences and the Risk of Lung Cancer

Within a mean follow-up of 11.6 years from baseline until the next follow up measurement, in which 173 lung cancer cases were identified, the mean BMI (-0.58 kg/m^2^, 95% CI: -0.62 – -0.53), WC (-2.79 cm, 95% CI: -3.00 – -2.58), and WHR (-0.016 95% CI: -0.019 – -0.014) decreased, whereas the ABSI mean (0.0350 95% CI: 0.0348 – 0.0352) increased. **Table 5** provides the hazard ratios for the associations between the mean differences of BMI, WC, WHR, and ABSI at baseline and follow-up with the risk of lung cancer. None of the associations between the mean differences at baseline and follow-up for BMI, WC, WHR, and ABSI with the risk of lung cancer were significant.

**Table 5:** Hazard ratio’s (95% CI) for the mean differences of BMI (kg/m^2^), WC (cm), WHR, and ABSI between baseline and follow-up with the risk of lung cancer.

	Number of LC cases	Model 1 HR, 95% CI	Model 2 HR, 95% CI
**Body Mass Index**			
Continuous	173	0.99 (0.89–1.09)	1.00 (0.91–1.10)
**Waist circumference**			
Continuous	173	1.00 (0.98–1.02)	1.01 (0.98–1.03)
**Waist-to-Hip Ratio**			
Continuous	173	1.03 (0.89–1.19)	1.02 (0.86–1.20)
**ABSI**			
Continuous	173	0.99 (0.86–1.15)	0.92 (0.77–1.10)

HR, Hazard Ratio; CI, Confidence Interval; LC, lung cancer; ABSI, A Body Shape Index.

Model 1: Adjusted for sex and age.

Model 2: Adjusted for sex, age, cohort ((RS-I/RS-II/RS-III), physical activity (continuous), alcohol intake (continuous), pack-years (continuous), education (high/low), WCRF adherence score (continuous), and diabetes mellitus (yes/no). WC and WHR were additionally adjusted for difference in BMI between baseline and follow-up. Thereby, all hazard ratios were adjusted for their own baseline measurement to correct for regression to the mean (35).

### Additional Analyses

**Figure 1:**
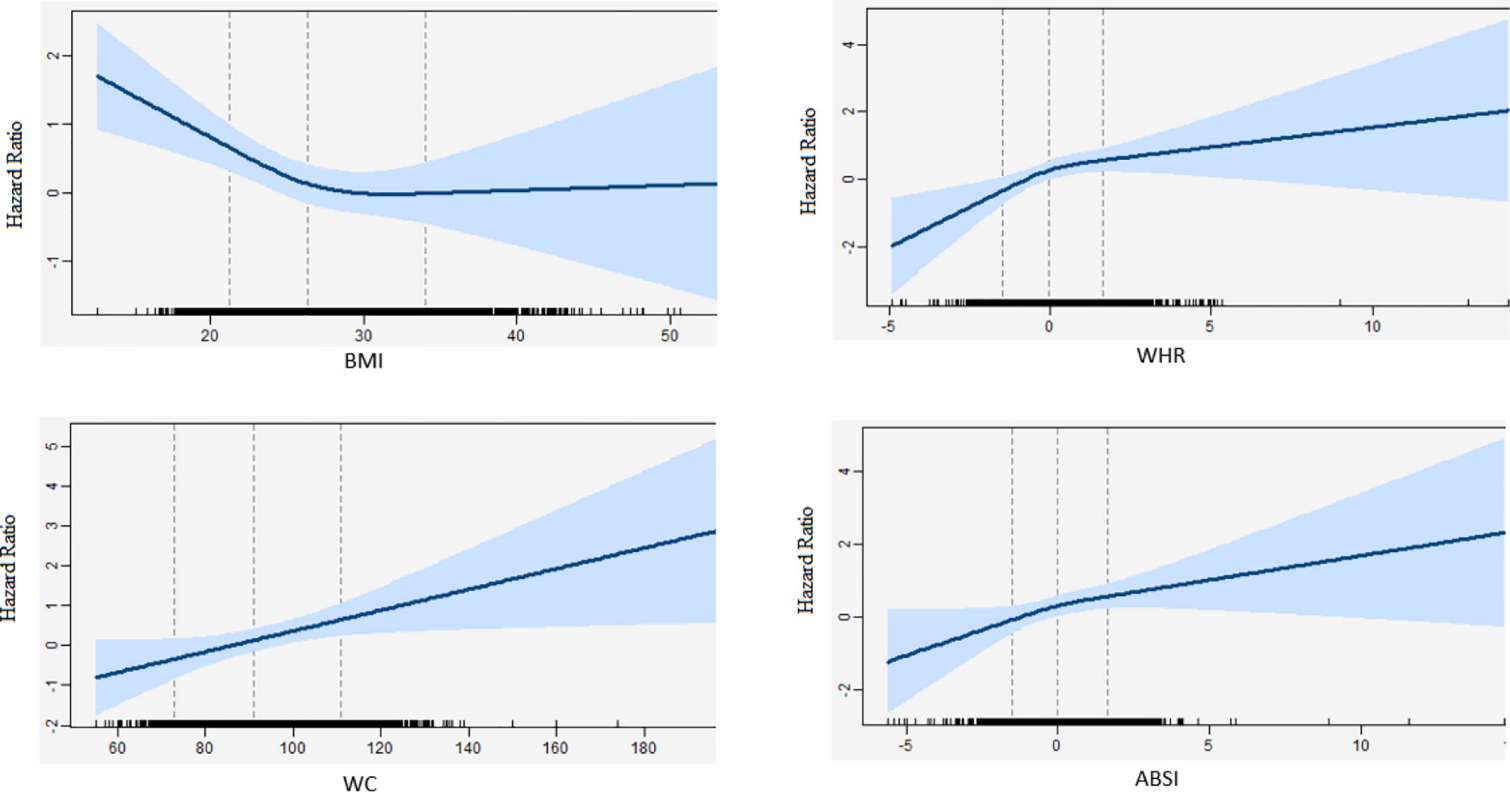
Natural cubic splines in a standard cox-regression model with knots at 22, 27, 34 and 72, 92, 112, and -1.5, 0, 1.5 and -1.5, 0, 1.5 for BMI, WC, WHR, and, ABSI respectively. The blue line represents the hazard ratios for the risk of lung cancer with the corresponding 95% confidence interval. Hazard ratios were adjusted for sex, age, cohort (RS-I/RS-II/RS-III), physical activity (continuous), alcohol intake (continuous), pack-years (continuous), education (high/low), WCRF adherence score (continuous), and diabetes mellitus (yes/no). WC and WHR were additionally adjusted for BMI.

In all Cox regression models, there was no significant interaction between follow-up time and BMI, WC, WHR, and ABSI. Therefore, the proportional hazard assumption was met for all associations. No significant interactions were observed between BMI, WC, WHR, and ABSI with smoking status and sex (data not shown). Furthermore, no significant interactions were found for BMI, WC, WHR, and ABSI with pack years of smoking **(**data not shown). **Figure 1** **s**hows the relation between 1-unit increase of BMI, WC, WHR, and ABSI with the risk of lung cancer for 9689 participants, including 319 lung cancer cases. None of the Likelihood-ratio (LR) tests between WC (p = 0.99, df =1), WHR (p = 0.09, df =1) or ABSI (p = 0.46, df =1) and the risk of lung cancer suggested a non-linear relationship. In contrast, the LR test between BMI (p = 0.04, df =1) and the risk of lung cancer did suggest a non-linear relationship. More specifically, the inverse association between BMI and lung cancer risk was only linear in non-obese participants whereas the curve flattened in obese participants.

## Discussion

In this prospective cohort study, we found that BMI was inversely associated with the risk of lung cancer. In contrast to BMI, an increased WC, WHR, and ABSI were associated with a higher risk of lung cancer. Additionally, we found evidence regarding a non-linear association between BMI and the risk of lung cancer, which indicated that the inverse association between BMI and lung cancer risk was only present in non-obese participants, whereas an increase in WC, WHR, and, ABSI showed a linearly higher risk of lung cancer.

Our results showed that a higher BMI was associated with a reduced risk of lung cancer which is in line with two meta-analyses (36, 37) and two pooled analyses (9, 38). Although these results seem to be consistent, this inverse association is in conflict with the hypothesis that suggests that obesity is a risk factor for lung cancer (39, 40). Residual confounding by smoking and reverse causation are proposed explanations for this inverse association (41, 42). Interestingly we found that the inverse association persisted after excluding lung cancer cases during the first 5, 7.5, and 10 years of follow-up. Our findings are in agreement with several other studies that attempted to address the role of reverse causation by excluding the lung cancer cases during the first years of follow-up. However, two other studies suggested that the strength of association between BMI and the risk of lung cancer diluted after excluding lung cancer cases during the first years of follow-up, indicating that reverse causation might play a role (10, 11). However, these two studies included mainly women, which can explain the different results because survival and clinical presentation for lung cancer differs among men and women (43). We also assessed the association between change in BMI and the risk of lung cancer between baseline and follow-up. Nonetheless, we did not find a significant association between a difference in BMI and the risk of lung cancer over time, which contradicts the role of reverse causation. Additionally, we assessed whether the relationship between BMI and the risk of lung cancer had a non-linear shape. We found evidence for a non-linear association between BMI and the risk of lung cancer suggesting that the inverse association only applies for participants without obesity. Previous studies showed that obesity is unbeneficial for developing lung cancer (20, 38), which could flattened the BMI-lung cancer curve for this group in our population.

An explanation for the inverse association between BMI and the risk of lung cancer in those without obesity may come from residual confounding by smoking. In the current study we found no association between BMI and the risk of lung cancer among never, ever, or current smokers. Several other studies which stratified the association for smoking found similar results (15–19). However, similar to our study, these studies had a relatively small number of lung cancer cases because lung cancer is quite rare in subjects without a history of smoking. Studies with larger sample sizes showed contrary results: a recent meta-analysis of 19 studies including 15 million never smokers and a pooled analysis of 12 studies containing almost 700,000 never smokers, showed that the inverse association also persisted in never smokers (9, 20). Thus, although the results in this study cannot fully rule out the presence of residual confounding by smoking, on the basis of our results combined with previous meta-analyses, smoking may not fully explain the inverse association between BMI and the risk of lung cancer.

An alternative biological explanation for the inverse association between BMI and the risk of lung cancer can be the storage of common environmental genotoxicants in fat tissue. Lipophilic genotoxicants such as polycyclic aromatic hydrocarbons (PAHs) that are derived from smoking or occupational exposure influence the development of lung cancer through DNA damage (44). Previous work has suggested that an individual’s BMI or fat tissue influences the metabolism of PAHs, in which lower levels of PAH-DNA adducts are found in the circulation of obese individuals (45). This may imply that individuals with lower fat tissue may have a higher exposure to these lipophilic carcinogens.

Our study found that WC and WHR were positively associated with the risk of lung cancer, consistent with other studies (9, 21). However, other studies that investigated the association between WC and the risk of lung cancer found more controversial results. A prospective study among Chinese participants found an inverse association between WC and the risk of lung cancer (23). Unlike our study, this study did not correct the analyses for BMI, which can explain the contradictory results. Another prospective cohort study found no association between WC and the risk of lung cancer (22). Although WHR and WC are both measurements of abdominal obesity, the association with the risk of lung cancer seems only consistent for WHR studies (9, 21). These dissimilar results could be explained by the fact that WHR has a stronger correlation with visceral fat when compared to WC (46). Increased visceral fat is positively associated with the risk of cancer (47), as visceral fat promotes inflammation and insulin resistance and may therefore create a pro-tumorigenic environment (48).

The findings is this study showed that ABSI was positively associated with the risk of lung cancer. Although this is the first study that examined the association between ABSI and the risk of lung cancer, another study among 200 adults with overweight found an inverse association between ABSI and FFMI (Fat Free Mass Index) (27), indicating that a decreased ABSI is associated with a higher FFMI when compared to higher ABSI values. A low FFMI may be an indicator for a higher risk of lung cancer as progressive loss of muscle mass is the main characteristic of lung cancer cachexia (49). However, since we focussed on incident lung cancer and did not find indications of reverse causality, our results on ABSI may not be explained by lung cancer cachexia. Another study among elderly found that in men, ABSI was positively associated with FM (Fat Mass) and thereby negatively associated with FFM (Fat Free Mass) (50). This study suggests that the positive association between ABSI and the risk of lung cancer may be dedicated to an increased FM, and may therefore support the hypothesis of body fatness and in particular abdominal obesity as a risk factor for lung cancer (50). Moreover, ABSI also shows a positive correlation with visceral fat (51). As mentioned earlier, increased visceral fat is associated with cancer progression (47) and may create a pro-tumorigenic environment (48). Therefore, more research is needed to identify the specific roles of visceral adiposity and FM and FFM in the relation to incident lung cancer as the VAT/SAT (Visceral and Subcutaneous Adipose Tissue) ratio is an independent predictor for non-small-cell lung cancer (52).

Although we found associations between baseline BMI, WC, WHR, and ABSI with the risk of lung cancer, we found no association for the mean differences between baseline and follow-up of BMI, WC, WHR, and ABSI with the risk of lung cancer. Another prospective cohort study among 8000 Dutch participants found that a moderate short-term annual decrease (-0.10 – -0.50 kg/m^2^/year) of BMI, was associated with a reduced risk of lung cancer in women (53). However, due to a lack of available data, this study could not fully correct the association between BMI and the risk of lung cancer for physical activity and alcohol consumption, both important confounders for the association between obesity and the risk of lung cancer. The non-significant results in this study can be explained in multiple ways. First, it should be mentioned that within the current study, anthropometric measures become less reliable during ageing due to shrinking and changes in body composition (i.e. loss of fat free mass) (54) and could have influenced the non-significant results because lung cancer has a long latency period. Secondly, as people become older, it is more likely that low body mass is reflected by a low muscle mass instead of a low fat mass (55, 56). This implies that BMI, WC, WHR, and ABSI may give an underestimation of the (abdominal) fat mass in a person. Consequently, these anthropometric measurements of body size and body shape become less valid. Therefore, future studies including larger sample sizes, longer years of follow-up, data on changes of covariates over time, and measurements that can distinguish fat and muscle mass, are needed to elucidate the role of body size and body shape over time in an appropriate manner.

An important strength of this study is that all anthropometric measurements are performed by trained staff in a special designed research centre according to a consistent protocol. Therefore, the chance of information bias in this study is minimal when compared to other studies (57). A second strength is the follow-up of 25 years. The Rotterdam Study has one of the longest follow-up in years when compared to other studies, which allowed us to assess the role of reverse causation over a longer time period.

This study has also some limitations. First, the number of incident lung cancer cases in this study was relatively low. As a result we could not perform extensive subgroup analysis since the association between obesity and lung cancer may differ for tumour histological type, ethnic origin, and sex (9). For example, non-smokers develop more often adenocarcinomas and different risk factors (e.g. genetic as well as environmental) can play a role (58). Furthermore, we were unable to conduct sensitivity analyses on the basis of COPD at baseline because of the small number of cases in our cohort. Despite the fact that only 19 lung cancer cases had COPD, the results preclude any conclusions regarding specific groups that may be more or less vulnerable to the consequences of (abdominal) obesity on lung cancer. Due to a lack of detailed data, we were also unable to correct our analysis for metabolic dysfunctions at baseline with the exception of diagnosis of type 2 diabetes. As metabolic dysfunctions are associated with central adiposity as well as lung cancer risk (59), the influence of early stage metabolic disturbances cannot be ruled out. Further, we did not have detailed measures on FFM specifically (eg. By DXA) to confirm that the association between ABSI and lung cancer is explained by sarcopenia, which has been found to be a predictor for lung cancer prognosis (60) as well as metabolic dysfunction (61). A second limitation was that the covariates in this study were only measured at baseline. The analysis of the mean differences between baseline and follow-up was therefore only adjusted for baseline covariables. Important covariates such as smoking and physical activity may have changed during follow-up and could have influenced our results. Additionally, these important covariables were self-reported, which usually leads to an overestimation of physical activity (61) and an underestimation of alcohol consumption (62). A third limitation is that the results in this study were mainly based on a population of traditionally Dutch origin. A pooled analysis found that the association between BMI and lung cancer was stronger among blacks when compared to whites (9). Hence, we may have underestimated the effect in our study when compared to studies which contained more diverse ethnic origins.

In conclusion, we found an inverse association between BMI and the risk of lung cancer. In contrast, WC, WHR and ABSI are positively associated. We found evidence that reverse causation may serve as an explanation for the inverse association between BMI and lung cancer. Future research with larger sample sizes should focus on the role of visceral fat and the distinction between fat mass from fat free mass, using more advanced measuring tools to get a better understanding of the underlying biological mechanisms.

## Data Availability Statement

The datasets presented in this article are not readily available because sharing of individual participant data was not included in the informed consent of the study, and there is a potential risk of revealing participants' identities. Requests to access the datasets should be directed to data manager Frank J.A. van Rooij (ln.cmsumsare@jioornav.f).

## Ethics Statement

The studies involving human participants were reviewed and approved by Medical Ethics Committee of the Erasmus MC. The patients/participants provided their written informed consent to participate in this study.

## Author Contributions

FA: writing of the manuscript, data analyses, revised the comments of the co-authors. RR: data acquisition, reviewing manuscript. MM: data acquisition, reviewing manuscript. LL: data acquisition, reviewing manuscript. BS: participated in setting up the study design, data acquisition, reviewing manuscript. JK-J: participated in setting up the study design, data acquisition, supervision of first author, critically reviewing the manuscript. All authors contributed to the article and approved the submitted version.

## Funding

The Rotterdam Study is supported by Erasmus University Medical Center and Erasmus University, Rotterdam, the NWO; the Netherlands Organization for Health Research and Development; the Research Institute for Diseases in the Elderly; the Netherlands Genomics Initiative; the Ministry of Education, Culture and Science; the Ministry of Health, Welfare and Sports; the European Commission (DG XII); and the Municipality of Rotterdam. The funders played no role in the study design or in data collection and analysis. The authors had final responsibility for design and conduct of the study, collection, management, analysis, and interpretation of the data, and preparation, review or approval of the manuscript.

## Conflict of Interest

The authors declare that the research was conducted in the absence of any commercial or financial relationships that could be construed as a potential conflict of interest.
